# Can habits and behaviors predict colonization by community-associated MRSA in patients admitted to a Brazilian hospital?

**DOI:** 10.1590/S1678-9946202466031

**Published:** 2024-05-13

**Authors:** Marcos Vinicius de Barros Pinheiro, Fernanda Sampaio Cavalcante, Dennis de Carvalho Ferreira, Ana Carolina Fonseca Guimarães, Adriana Lúcia Pires Ferreira, Claudia Regina da Costa, Kátia Regina Netto dos Santos, Simone Aranha Nouér, Infection Control Group HUCFF/UFRJ, Ana Pereira Rangel, Anna Carla Castiñeiras, Christiany Moçali Gonzalez, Joana Freire, Luiz Felipe Guimarães, Raquel Batista

**Affiliations:** 1 Universidade Federal do Rio de Janeiro, Faculdade de Medicina, Programa de Pós-Graduação em Doenças Infecciosas e Parasitárias, Rio de Janeiro, Rio de Janeiro, Brazil; 2 Universidade Federal do Rio de Janeiro, Instituto de Ciências Médicas, Departamento de Clínica Médica, Macaé, Rio de Janeiro, Brazil; 3 Universidade do Estado do Rio de Janeiro, Rio de Janeiro, Rio de Janeiro, Brazil; 4 Universidade Federal do Rio de Janeiro, Instituto de Microbiologia Paulo de Góes, Departamento de Microbiologia Médica, Rio de Janeiro, Rio de Janeiro, Brazil; 5 Universidade Federal do Rio de Janeiro, Hospital Universitário Clementino Fraga Filho, Comissão de Controle de Infecção Hospitalar, Rio de Janeiro, Rio de Janeiro, Brazil

**Keywords:** CA-MRSA, Colonization, Risk factor, Admission

## Abstract

This study aimed to identify factors associated with colonization by community-associated methicillin-resistant *Staphylococcus aureus* (CA-MRSA) in adult patients admitted to a Brazilian hospital. This is a cross-sectional study, in which patients underwent a nasal swab and were asked about hygiene behavior, habits, and clinical history. Among the 702 patients, 180 (25.6%) had *S. aureus* and 21 (2.9%) MRSA. The factors associated with MRSA colonization were attending a gym (OR 4.71; 95% CI; 1.42 – 15.06), smoking habit in the last year (OR 2.37; 95% CI; 0.88 – 6.38), previous hospitalization (OR 2.18; CI 95%; 0.89 – 5.25), and shared personal hygiene items (OR 1.99; 95% CI; 0.71 – 5.55). At the time of admission, colonization by CA-MRSA isolates was higher than that found in the general population. This can be an important public health problem, already endemic in hospitals, whose factors such as those associated with habits (smoking cigarettes) and behaviors (team sports practice and activities in gyms) have been strongly highlighted. These findings may help developing infection control policies, allowing targeting patients on higher-risk populations for MRSA colonization.

## INTRODUCTION

*Staphylococcus aureus* is one of the main human infectious agents. Almost 50% of healthy individuals harbor the bacteria in a persistent or intermittent state^1^. The epidemiology of MRSA has changed with the global emergence of community-associated MRSA (CA-MRSA) strains^2^. In addition to the widespread increase in *S. aureus* reservoirs, the emergence of CA-MRSA has hampered therapy in recent years^3^.

In Brazil, the lack of studies is a notable limiting factor in the knowledge and epidemiology of CA-MRSA among patients admitted to hospitals. These individuals may be admitted to hospitals, be subjected to surgeries, and previous decolonization could be considered; thus, this information would have an impact as an infection prevention measure. On the other hand, in health facilities where this pathogen is not endemic, the admission of colonized patients could introduce a new multidrug-resistant pathogen and cause its spread. To date, potential factors associated with infection or colonization by CA-MRSA are not fully established, making it difficult to prioritize a strategy based on higher-risk populations. This study aimed to identify the potential factors related to colonization by CA-MRSA in patients admitted to our teaching hospital in Rio de Janeiro, Brazil.

## MATERIALS AND METHODS

This is a randomized, cross-sectional, and epidemiological study based on patients admitted to the Hospital Universitario Clementino Fraga Filho (HUCFF) from Universidade Federal do Rio de Janeiro (UFRJ). This is a federal, public, tertiary, and teaching hospital. During the study period, the hospital presented 280 beds and about 8,000 admissions per year, including internal medicine and surgery, intensive care units, hematology, hemodialysis for chronic patients, and emergency wards. From May 2012 to June 2014, patient groups were analyzed to assess risk factors related to the admission of MRSA-colonized patients compared to noncolonized patients. The study was approved by the Research Ethics Committee of the Hospital Universitario Clementino Fraga Filho.

Inclusion criteria were hospitalized patients aged over 18 years, of both sexes, of all ethnic groups, in different health states, belonging to any social group, regardless of the use of antimicrobials, and with less than 72 hours of hospitalization. The acceptance was formalized by signing an informed consent form. Exclusion criteria were patients with reduced level of consciousness; cases in which sample collection for microbiological examination was not possible; laboratory investigations failed, with isolation of MRSA in a colonization or infection sample up to 12 months prior to the study; patients with swab collection that exceeded 72 h since hospital admission; and patients admitted to Intensive Care Units.

On average, 10 to 15 patients per day were eligible for the study. From them, five patients were randomly selected each day for the nasal swab collection. Interviews were conducted to obtain, among other information, epidemiological data, for which a standard form with open- and closed-ended questions was used, recording socioeconomic and demographic information, habits, routines, living environment, hospital data, and clinical information. Information about clinical history was complemented using the patients’ medical records. The information obtained from the medical records was used to calculate the Charlson score for comorbidities^4^. MRSA colonization or infection during hospitalization or earlier hospitalization were investigated using laboratory information from collected cultures, as part of routine care for selected patients, in the Bacteriology Laboratory. A standard electronic form used to record data (database) was created using the EpiInfo^®^ software (version 3.5.2, Centers for Disease Control and Prevention, Atlanta, GA, USA).

MRSA detection and isolation was performed via a nasal swab previously moistened in 0.9% saline solution, rubbed in rotational movements in the anterior region of both nostrils by healthcare professionals. The specimens were inoculated on mannitol salt agar (Oxoid, Basingstoke, United Kingdom). *S. aureus* isolates were previously identified and characterized for methicillin resistance^5^. Linear regression was used to analyze factors associations and the results were presented as Odds ratio (OR), with 95% confidence intervals (95% CI). The variables that presented p <0.2 in the univariate analysis were selected to compose the multivariate model. The inclusion of selected dependent variables established the multiple regression model using the Stepwise Backward method to compare the complete model with the reduced model and exclude the worst performing variable.

## RESULTS

In the study period, HUCFF registered 16,763 admissions, excluding admissions to the Intensive Care and Emergency Units. For the study, 702 patients were included and underwent nasal swab collection. Mean age was 48.4 years (SD = 16.12), mean body mass index (BMI) was 26.5, 51.7% (n = 363) were women, 54% (n = 379) declared to be White, 45.2% (n = 317) had completed secondary education, and 65.5% (n = 459) were residents of Rio de Janeiro municipality.

The main reason for hospitalization was surgical or surgery-related procedures (71%; n = 498), with 68% (n = 477) of them undergoing orthopedic surgeries. A total of 9.5% (n = 67) were transferred from other hospitals. Hospital length of stay ranged from one to 30 days, with a median of five days; 679 patients (96.7%) were discharged, 22 patients (3.1%) were transferred to the ICU, and one (0.2%) patient died. The Charlson score median was 1.0, in which 76.8% (n = 539) of the patients did not present any comorbidity. However, considering comorbidities, diabetes with insulin dependence was the most frequent (5.3%; n = 37).

Table 1 shows the univariate analysis of 702 admissions with 180 patients with *S. aureus* (both MSSA and MRSA) in relation to 522 patients without *S. aureus* colonization. The following associated factors were observed: animal-related activity (OR: 5.96; 95% CI: 1.47 – 24.10), use of illicit drugs (OR: 3.29; 95% CI: 1.55 – 6.97), active sex life (OR: 3.29; 95% CI: 1.55 – 6.97), smoking habit in the last year (OR: 1.49; 95% CI: 0.96 – 2.30), and having a Charlson score (<1) (OR: 1.49; 95% CI: 1.08 – 2.07).

**Table 1:** Univariate and multivariate analysis of potential factors associated with *S. aureus* colonization in 702 patients admitted in a public tertiary care hospital.

Potential associated factor	Yes	No	Univariated analysis			Multivariated analysis			

	N (%)	N (%)							
	

	180 (25.6)	522 (74.4)	OR	(95% CI)	P value	OR	(95% CI)		P value
Sex (Female)	87 (48.3)	256 (49)	1.38	0.57 – 3.34	0.46				
Age -mean	47.4	48.6							
Ethnicity (White)	111 (61.7)	268 (51.3)	1.01	0.58 – 1.78	0.94				
BMI – mean	26.3	26.4							
Complete secondary education	86 (47.7)	231 (44.2)	0.75	0.57 – 0.97	0.04				
Antibiotic use last 3 days	15 (8.3)	81 (15.5)	0.49	0.27 – 0.88	0.02				
Bed sharing	66 (36.7)	226 (43.3)	0.75	0.53 – 1.07	0.12				
Towel sharing	124 (68.9)	353 (67.6)	1.05	0.73 – 1.52	0.75				
Animal-related activity	6 (3.3)	3 (0.6)	5.96	1.47 – 24.1	0.01*	5.97	1.45	24.44	0.010*
Healthcare worker	13 (7.2)	46 (8.8)	0.8	0.42 – 1.52	0.51				
Pet at home or work	66 (36.7)	246 (47.1)	0.64	0.45 – 0.92	0.02				
Attending a gym	12 (6.6)	30 (5.7)	1.17	0.58 – 2.34	0.65				
Collective sport practice	9 (5)	29 (5.6)	0.89	0.41 – 1.92	0.77				
Use of illicit drugs	15 (8.3)	14 (2.7)	3.29	1.55 – 6.97	0.01*	3.05	1.42	6.55	0.004*
Tattoo^**^	17 (9.4)	47 (9)	1.05	0.58 – 1.88	0.86				
Active sex life	131 (7.7)	323 (61.9)	1.64	1.13 – 2.39	0.01*	1.50	1.02	2.19	0.030*
Smoking habit^**^	37 (20.7)	77 (14.7)	1.49	0.96 – 2.33	0.06*				
Family member with hospitalization history	19 (10.5)	76 (14.6)	0.69	0.4 – 1.1	0.18				
Physically dependent family member	15 (8.3)	38 (7.3)	1.15	0.62 – 2.15	0.63				
Hospitalization (≥3 days)^**^	53 (29.4)	202 (38.7)	0.66	0.45 – 0.9	0.02				
Use of antibiotics^**^	47 (26.1)	147 (28.2)	0.9	0.61 – 1.32	0.57				
Invasive procedure^**^	23 (12.7)	118 (22.6)	0.5	0.3 – 0.81	0.01				
Transferred from another institution	21 (11.6)	46 (8.8)	1.36	0.79 – 2.36	0.26				
Invasive intervention	66 (36.6)	190 (36.4)	1.01	0.71 – 1.43	0.95				
Underlying disease	102 (56.6)	346 (66.3)	0.66	0.47 – 0.94	0.02				
Charlson score (<1)	14 (7.7)	412 (78.9)	1.49	1.08 – 2.07	0.02*	1.53	1.10	2.13	0.010*

CI = confidence interval; BMI = body mass index; m: months*;* *Variable selected to compose the multivariate model; **in the last year.

The prevalence of MRSA colonization at admission was 2.9% (21/702) (95% CI: 1.96 – 4.53). Among the MRSA colonized patients, eight (38%) had surgical indication, being seven (33.3%) for orthopedic surgery and one (4.7%) for cardiac surgery. Table 2 shows the results from the analysis of potential factors associated with MRSA colonization. In the univariate analysis, the factor “attending a gym” showed statistical significance (OR: 3.98; 95% CI: 1.27 – 12.41). In the multivariate analysis, the risk factors associated with a higher chance of MRSA colonization were attending a gym (OR: 4.71; 95% CI: 1.42 – 15.06), smoking habit in the last year (OR: 2.37; 95% CI: 0.88 – 6.38), hospitalization for more than three days in the last year (OR: 2.18; 95% CI: 0.89 – 5.25), and sharing personal hygiene items (OR: 1.99; 95% CI: 0.71 – 5.55) (Table 2). Even though this last variable did not present statistical significance in the univariate analysis, it contributed to the final model.

**Table 2:** Univariate and multivariate analysis of potential factors associated with methicillin-resistant *S. aureus* colonization in 702 patients admitted to a public tertiary care hospital.

Potential associated factor	Yes	No	Univariated analysis			Multivariated analysis			

	N (%)	N (%)							
	
	21 (2.9)	681 (97.1)	OR	(95% CI)	P value	OR	(95% CI)		P value
Sex (Female)	12 (57.1)	350 (51.4)	0.7	0.29 – 1.7	0.44				
Age -mean	48.6	48.4							
Ethnicity (white)	11 (52.4)	368 (54)	1.04	0.6 – 1.8	0.87				
BMI – mean	26.4	26.4							
Complete secondary education	15 (71.4)	304 (44.6)	1.37	0.73 – 2.59	0.32				
Antibiotic use last 3 days	4 (19)	81 (15.5)	1.5	0.49 – 4.58	0.47				
Bed sharing	10 (47.6)	226 (43.3)	1.28	0.53 – 3.06	0.47				
Towel sharing	10 (47.6)	467 (68.6)	0.41	0.17 – 0.99	0.05*	1.99	0.71	5.55	0.09
Animal-related activity	1 (4.8)	8 (1.2)	4.22	0.5 – 35.27	0.18				
Healthcare worker	3 (14.3)	56 (8.2)	1.8	0.53 – 6.5	0.33				
Pet at home or work	7 (33.3)	305 (44.8)	0.61	0.24 – 1.54	0.3				
Attending a gym	4 (19)	38 (5.6)	3.98	1.27 – 12.41	0.02*	4.71	1.42	15.06	0.01*
Collective sport practice	3 (14.3)	35 (5.1)	3.07	0.86 – 10.93	0.08				
Use of illicit drugs	1 (4.8)	28 (4.1)	1.61	0.15 – 9	0.44				
Tattoo^**^	1 (4.8)	63 (9.3)	0.49	0.6 – 3.71	0.49				
Active sex life	14 (66.7)	440 (64.6)	0.79	0.29 – 1.7	0.45				
Smoking habit^**^	6 (28.6)	108 (15.9)	2.12	0.8 – 5.6	0.13	2.37	0.88	6.38	0.05*
Family member with hospitalization history	4 (19)	91 (13.4)	1.52	0.5 – 4.46	0.45				
Physically dependent family member	2 (9.5)	51 (7.5)	1.3	0.29 – 5.73	0.73				
Hospitalization (≥3 days)^**^	11 (52.4)	244 (35.8)	1.97	0.82 – 4.7	0.13	2.18	0.89	5.25	0.06
Use of antibiotics^**^	5 (23.8)	189 (27.7)	0.81	0.29 – 2.25	0.69				
Invasive procedure^**^	2 (9.5)	139 (20.4)	0.41	0.09 – 1.78	0.23				
Transferred from another institution	1 (4.8)	66 (9.7)	0.46	0.06 – 3.52	0.46				
Invasive intervention	9 (42.9)	247 (36.3)	0.43	0.09 – 1.83	0.24				
Underlying disease	2 (9.5)	437 (64.2)	0.61	0.25 – 1.46	0.27				
Charlson score (<1)	9 (42.9)	525 (77.1)	1.34	0.6 – 2.29	0.46				

CI = confidence interval; BMI = body mass index; m = months*;* *Variable selected to compose the multivariate model; ^**^in the last year.

Regarding MRSA-colonized patients in the admission, the average age was 49.7 years old and the median for the hospitalization period was 14 days. Most of them admitted for surgical procedures (66%). At least one baseline disease was common in 61.9% of patients and the median Charlson comorbidity score was 1.0. The molecular analysis of the 21 MRSA isolates showed that 15 (71.4%), three (14.3%), and two (9.5%) presented SCC*mec* IV, SCC*mec* II, and SCC*mec* III, respectively (Table 1).

Among the patients enrolled in this study, three (14.3%) developed infection after admission, one by MRSA. This patient was 67 years old, with cardiovascular conditions, and hypertensive, admitted to elective cardiac surgery (myocardial revascularization). He received routine surgical prophylaxis with no effectiveness against MRSA. Then, 10 days after surgery, developed mediastinitis, caused by MRSA. The assistant team opted to administer teicoplanin to treat this infection. After two surgical interventions and without confirmation of bone involvement, the patient was discharged from the hospital 30 days after the surgery. During the follow-up period, for more than a year in the cardiac surgery outpatient clinic, the patient did not present any complications until the last return and medical discharge.

## DISCUSSION

To our knowledge, this was the only study conducted in the last decade that assessed the prevalence of MRSA colonization in patients admitted to a Brazilian hospital and explored the factors associated with colonization. Here, the prevalence of *S. aureus* and MRSA colonization was 25.6% and 2.9%, respectively. Rates of *S. aureus* was similar to those recently detected in Brazil for urban outpatients, which was found to range from 17% to 33% when healthcare professionals and children were excluded^6-10^.

Related to MRSA prevalence, although no recent studies have evaluated the rates of this pathogen among hospitalized patients, our data were very similar to a previous survey conducted in 2018 that found 2.3% MRSA carriers in a sample with 300 patients of Sao Paulo city who attended to a first aid emergency and infectious diseases or dermatology ambulatory^8^. On the other hand, the prevalence found was higher than the detected in the Brazilian urban population (0.9%)^7^, but lower than that found for individuals with comorbidities, such as diabetes (4.8%)^11^ and HIV (4.4%)^12^. We hypothesize that the prevalence of MRSA among hospitalized adults is correlated with the proportion of individuals suffering from chronic diseases since we found a median Charlson score of 1.0 in patients, with more than 75% of them not presenting comorbidities.

Moreover, we found that attending a gym was a risk factor for MRSA colonization. Gym equipment used by multiple users without proper hygiene can be a source of *S. aureus*. Markley *et al*.^13^ found methicillin-susceptible *S. aureus* (MSSA) strains in 10% of the isolates collected from equipment and environments at a gym in the USA. For the authors, just as MSSA remained on surfaces, MRSA isolates can also remain in these environments. Likewise, infection is commonly seen in team sports via sharing uniforms or towels without proper hygiene, coupled with physical contact. This study showed that 14% of patients played team sports and were MRSA colonized. The association between team sport with physical contact and MRSA colonization was first observed in 2005^14^ on a college football team in Atlanta (USA). It was found that 8% of the athletes were MRSA-colonized, in addition to those with MRSA skin and soft tissue infections. Furthermore, another study with university athletes conducted in the USA found that contact sports athletes had a higher risk of MRSA colonization over time^15^.

Another factor associated with MRSA colonization was the sharing of personal hygiene items. It is possible that the bacteria spread by indirect contact via objects previously touched by colonized individuals such as towels, razors, and nail clippers, when they penetrate in the lesioned skin. In this study, we found that the colonization chance was almost twice as high as those who did not share personal hygiene items. A recent study conducted with 127 dermatology outpatients in Sao Paulo city, Brazil, found a positive association between MRSA and the sharing of personal objects (shaver, soap, and bath sponges)^8^. The fitted model showed a probability of identifying 79% of colonized patients based on the potential factors associated with MRSA colonization.

In this study, smoking habit was a statistically significant risk factor for MRSA carriage. McEachern *et al.* ^16^ reported cigarette smoke may modulate the virulence of these strains, since MRSA isolates exposed to cigarette smoke extract are more resistant to macrophage killing and show reduced susceptibility to cell lysis and antimicrobial peptide killing. They also stated that cigarette smoking increases the expression of genes linked to changes in the surface of MRSA cells.

There is controversy as to whether universal MRSA screening on admission significantly affects infection rates and reduces transmission, or whether it is costly when compared with risk factor-based screening^17^. In this context, Roth *et al*.^18^ showed that MRSA screening based on risk factors associated with colonization presents a lower cost of hospital surveillance than universal screening. *S. aureus* screening for colonized patients was later adopted for specific procedures such as cardiology, orthopedics, and solid organ transplantation as a measure to reduce surgical infections^19^. Decolonization of these patients before surgery reduces approximately 80% of infections and has been recommended in patients undergoing cardiothoracic and orthopedic surgeries^20^.

As a limitation of this work, we highlight the period in which our study was conducted. However, studies published in recent years with other populations indicate that our findings remain up to date. Furthermore, as no Brazilian work has been conducted on this topic in the last decade, this study is important to guide infection control policies in Brazilian hospitals.

## CONCLUSION

In conclusion, our study strongly highlighted factors associated with habits (smoking cigarettes) and behaviors (practicing of team sports and activities in gyms) in previously MRSA-colonized patients admitted to a hospital in Rio de Janeiro city. Therefore, systematic and coordinated infection control policies are necessary and essential to evaluate the best intervention strategy used inside and outside the hospital, such as identifying patients at a higher risk for MRSA colonization.

## INFECTION CONTROL GROUP HUCFF/UFRJ

Ana Pereira Rangel, Anna Carla Castiñeiras, Christiany Moçali Gonzalez, Joana Freire, Luiz Felipe Guimarães, Raquel Batista.
